# Metabolomics biomarkers to predict acamprosate treatment response in alcohol-dependent subjects

**DOI:** 10.1038/s41598-017-02442-4

**Published:** 2017-05-31

**Authors:** David J. Hinton, Marely Santiago Vázquez, Jennifer R. Geske, Mario J. Hitschfeld, Ada M. C. Ho, Victor M. Karpyak, Joanna M. Biernacka, Doo-Sup Choi

**Affiliations:** 1 Department of Psychiatry and Psychology, Mayo Clinic College of Medicine, Rochester, Minnesota 55905 USA; 2 Department of Molecular Pharmacology and Experimental Therapeutics, Mayo Clinic College of Medicine, Rochester, Minnesota 55905 USA; 3 Neurobiology of Disease Program, Mayo Clinic College of Medicine, Rochester, Minnesota 55905 USA; 4 University of Puerto Rico School of Medicine, San Juan, Puerto Rico; 5 Department of Biomedical Statistics and Informatics, Mayo Clinic College of Medicine, Rochester, Minnesota 55905 USA; 6 Psychiatry and Mental Health Service, Sotero Del Rio Hospital, Santiago, Chile

## Abstract

Precision medicine for alcohol use disorder (AUD) allows optimal treatment of the right patient with the right drug at the right time. Here, we generated multivariable models incorporating clinical information and serum metabolite levels to predict acamprosate treatment response. The sample of 120 patients was randomly split into a training set (*n* = 80) and test set (*n* = 40) five independent times. Treatment response was defined as complete abstinence (no alcohol consumption during 3 months of acamprosate treatment) while nonresponse was defined as any alcohol consumption during this period. In each of the five training sets, we built a predictive model using a least absolute shrinkage and section operator (LASSO) penalized selection method and then evaluated the predictive performance of each model in the corresponding test set. The models predicted acamprosate treatment response with a mean sensitivity and specificity in the test sets of 0.83 and 0.31, respectively, suggesting our model performed well at predicting responders, but not non-responders (i.e. many non-responders were predicted to respond). Studies with larger sample sizes and additional biomarkers will expand the clinical utility of predictive algorithms for pharmaceutical response in AUD.

## Introduction

Alcohol use disorder (AUD) is a heterogeneous and complex psychiatric disorder that affects 4–5% of the world population^1^. It is often associated with other substance disorders as well as other psychiatric disorders and represents a substantial worldwide economic burden^2, 3^. The heterogeneity of patient populations with AUD as well as associated comorbidities complicate treatment outcome^4, 5^. This is underscored by the fact that the number needed to treat (NNT) to prevent one person from returning to any alcohol consumption for acamprosate is 12 and for naltrexone is 20 while studies of disulfiram do not support an association with preventing return to heavy drinking^6^. This heterogeneity in AUD suggests that a single drug will not work for all patients^7^. Although research should focus on the development of novel medications for the treatment of AUD, perhaps the best way to optimize the currently available FDA approved medications (disulfiram, naltrexone and acamprosate) as well as any new drugs that are approved in the future, is to identify biomarkers of treatment response in an effort to develop a personalized medicine approach in AUD.

The goal of personalized medicine is to identify a single or group of measurable biological factors that are capable of identifying which patients have the highest likelihood of responding to a particular treatment intervention^8, 9^. Personalized medicine in alcohol use disorder is still at its early stages but has first focused on pharmacogenomics biomarkers of treatment response^10, 11^. A single nucleotide polymorphism (SNP) in the μ-opioid receptor gene (*OPRM1*) was found to be associated with naltrexone treatment response^12–14^. However, when a prospective study was conducted to determine whether this particular SNP in OPRM1 predicts naltrexone response, the authors were unable to conclude that the Asp40 allele of OPRM1 moderates response to naltrexone treatment^15^. More traction has been gained when examining pharmacogenomics makers of acamprosate treatment response. Several independent groups have found associations between SNPs in a GABA_A_ receptor subunit gene [*GABRB2* ^16^], a transcription factor for atrial natriuretic peptide [*GATA4* ^17^] and an NMDA glutamate receptor subunit gene [*GRIN2B* ^18^] with acamprosate treatment response. In addition, in animal studies, mice that lack type 1 equilibrative nucleoside transporter [*ENT1* ^19^] or have a mutation in period 2 [*PER2* ^20^] showed reduced alcohol consumption in response to acamprosate treatment while no effect was observed in wild-type mice. However, prospective studies to evaluate the predictive power of these pharmacogenomics biomarkers have not been performed.

Metabolomics can identify pretreatment or response-to-treatment metabolotypes that can be associated with the outcome of a particular pharmacological intervention. Thus, metabolic signatures that are associated with a positive or negative treatment response could be used to predict patient treatment outcomes^21–23^. Several studies have used metabolomics to identify baseline pre-treatment metabolic signatures that are associated with treatment response to sertraline in depression^24, 25^, and to antipsychotics in schizophrenia^26, 27^. In addition, we have used a metabolomics method to identify baseline metabolites that are associated with treatment response to acamprosate in patients with AUD^28^.

In this study, we aimed to investigate the potential utility of multivariable models incorporating baseline metabolic and clinical biomarkers to identify patients that have the highest likelihood of a positive treatment response (no alcohol consumption for at least 3 months) to acamprosate. Overall, the identification of biomarkers and the development of predictive models that are capable of predicting therapeutic response to pharmacological agents for treatment of AUD could help physicians to determine which medication to prescribe for individual AUD patients, enabling personalized medicine in AUD. To our knowledge, this is the first study describing the use of predictive models incorporating both metabolomic and clinical biomarkers to determine treatment response to acamprosate.

## Results

### Differences in baseline demographics, alcohol use history and clinical features between responders and non-responders

Prior to building multivariable predictive models of acamprosate response, we compared baseline demographic and clinical features between responders and non-responders. There was no difference in age at time of consent or race between responders and non-responders; however, there was a higher proportion of males in the responder group (*P* = 0.029). In addition, there was marginally significant evidence suggesting that responders reported a heavier drinking pattern (average number of drinks per drinking day) in the 3 months prior to entering the study compared to non-responders (*P *=0.059).There was no difference in average number of drinks per drinking day in the past month before entering the study or the average number of days sober in the past 3 months before entering the study. Baseline PACS scores were higher in non-responders compared to responders (*P *<0.001). The baseline activity of glutamine synthetase and baseline GGT levels were similar between the groups (Table 1).

**Table 1:** Comparison of demographics, drinking history, and clinical characteristics between responders and non-responders.

Measure	Responders (*n* = 71)		Non-responders (*n* = 49)		*P* Value
	Mean or *n*	SD or %	Mean or *n*	SD or %	
*Demographics*					
Age at consent	45.9	10.5	44.7	11.6	0.549
Gender (male)	54	76.1	28	57.1	0.029
Race (Caucasian)	67	94.4	43	87.8	0.198
No. of drinks/day (3 months prior)	11.9	9.9	9.0	4.7	0.059
No. of drinks/day (1 month prior)	10.4	12.7	7.5	5.5	0.137
No. of days since last drink	27.7	24.2	20.9	19.7	0.157
*Clinical Characteristics*					
Baseline PACS Score	11.6	7.1	16.4	8.6	<0.001
GS activity	4.3	2.7	3.8	1.9	0.660
GGT	82.1	88.1	62.0	70.4	0.737

GS: glutamine synthetase; GGT: glutamyl transpeptidase; PACS: Pennsylvania Alcohol Craving Scale; SD: Standard Deviation.

### Differences in baseline serum metabolites between responders and non-responders

We evaluated the level of 36 serum amino acids and amino acid derivatives in responders and non-responders to 3 months of acamprosate treatment (Table 2). We found that levels of 6 metabolites (glutamate, ammonia, 1-methylhistidine, taurine, aspartate, and threonine) were different between responders and non-responders (uncorrected *P* < 0.05).

**Table 2:** Baseline serum metabolite levels in responders and non-responders to 3-month acamprosate treatment in the training sample.

Measure	Responders (*n* = 71)		Non-responders (*n* = 49)		*P* Value
	Mean or *n*	SD or %	Mean or *n*	SD or %	
*Serum Metabolite Levels*					
1-Methylhistidine	7.50	5.87	13.14	17.19	0.023
3-Methylhistidine	4.36	2.92	4.47	2.20	0.822
α-aminoadipic acid	1.10	0.68	0.83	0.60	0.027
α-amino-n-butyric	13.63	5.33	15.57	7.03	0.089
Alanine	400.38	79.59	389.94	92.85	0.511
Ammonia	34.40	17.66	26.17	13.22	0.007
Arginine	100.39	23.52	98.41	24.69	0.659
Asparagine	61.20	14.97	61.86	17.51	0.825
Aspartate	19.00	10.14	13.00	6.90	<0.001
β-alanine	5.47	2.05	6.30	5.18	0.225
β-aminoisobutyric acid	0.89	0.50	0.91	0.58	0.865
Citrulline	27.86	8.16	29.81	9.55	0.233
Cysthationine	1.10	1.05	0.92	0.99	0.387
Cystine	77.64	21.48	81.03	20.75	0.391
Ethanolamine	8.74	2.39	7.93	2.87	0.096
Glutamate	31.71	16.14	22.66	9.98	<0.001
Glutamine	741.99	157.06	760.09	208.16	0.589
Glycine	283.35	93.11	261.24	63.52	0.151
Histidine	90.74	25.94	91.11	19.61	0.933
Hydroxylysine	1.35	0.91	1.13	0.82	0.184
Hydroxyproline	17.06	8.21	14.99	8.60	0.187
Isoleucine	64.72	21.46	66.02	21.86	0.747
Leucine	132.23	40.55	130.75	38.45	0.841
Lysine	153.08	45.09	157.85	60.55	0.622
Methionine	20.23	5.82	21.37	7.47	0.350
Ornitine	94.43	38.11	83.39	38.21	0.122
Phenylalanine	69.98	17.69	65.87	15.15	0.188
Phosphoethanolamine	1.95	1.69	1.42	1.21	0.069
Proline	220.76	62.32	221.87	80.47	0.932
Sarcosine	1.19	0.55	1.27	0.79	0.557
Serine	113.12	26.58	108.07	28.73	0.325
Taurine	171.69	94.39	131.01	51.67	0.007
Threonine	113.63	23.25	130.04	43.70	0.009
Tryptophan	60.12	15.74	63.33	18.62	0.310
Tyrosine	70.60	21.15	70.67	27.07	0.986
Valine	232.19	65.14	225.92	55.37	0.583

SD: Standard Deviation.

### Predictive model development using the training samples and evaluation of performance in the test samples

**Figure 1:**
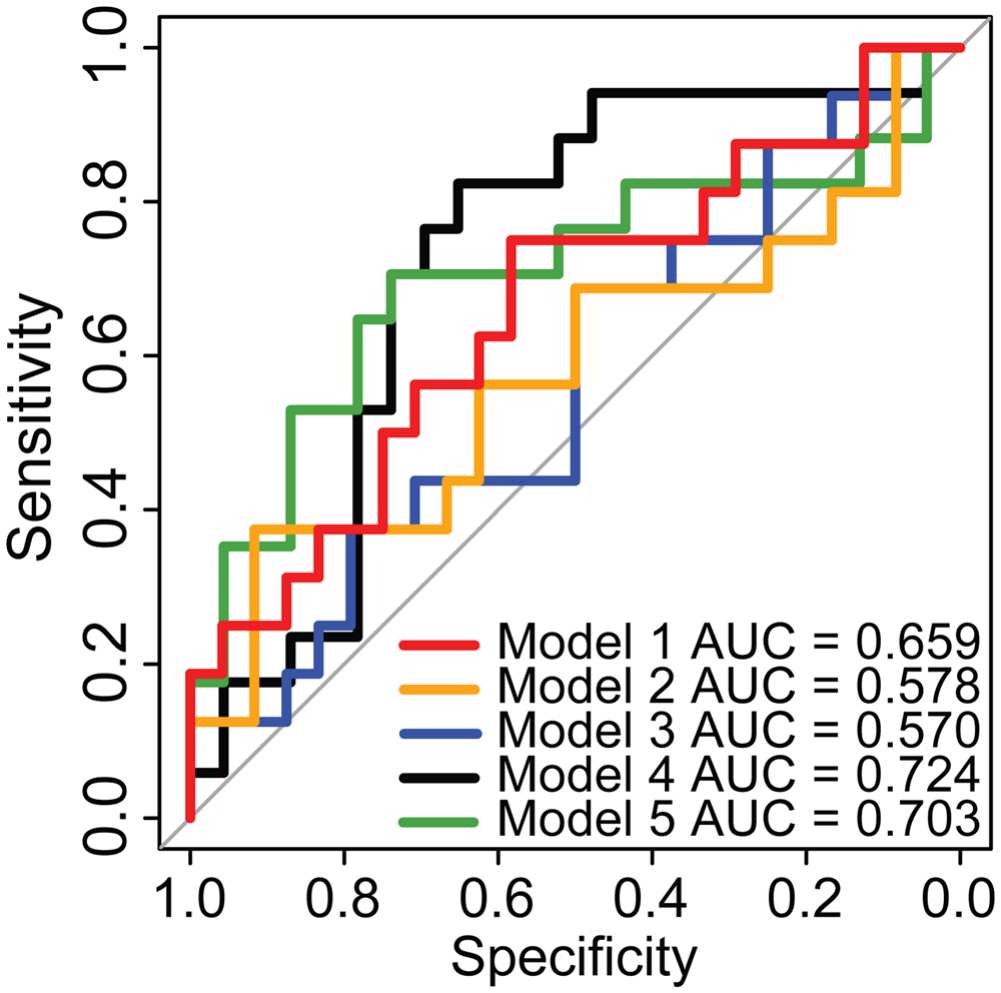
Receiver operating characteristic (ROC) curves illustrating the ability of the Lasso penalized selection models to predict acamprosate treatment response in the test set for each of the 5 random splits of the data.

Table 3 shows the best model, based on five-fold cross-validation, for prediction of acamprosate treatment response in each of the 5 random training subsets. PACS score and aspartate were the most consistently selected predictors, being included in all five predictive models. 1-Methylhistidine, ammonia, taurine, threonine, and male gender were all included in at least two predictive models. Table 4 presents the performance of the models in the five testing samples. The area under the ROC curve (AUC) in the test set varied between the five random data splits, ranging from 0.570 to 0.724 (mean 0.647) (Fig. 1). In the test samples, the models predicted acamprosate treatment response with a sensitivity ranging from 0.708 to 1.000 (mean sensitivity = 0.833) and a specificity ranging from 0.059 to 0.500 (mean specificity = 0.310).

**Table 3:** Multivariable logistic regression model variables selected by Lasso penalized selection method.

Predictor	Model 1	Model 2	Model 3	Model 4	Model 5
Intercept	−0.601	0.558	0.643	0.252	0.340
Baseline PACS	−0.015	−0.061	−0.057	−0.005	−0.023
Aspartate	0.026	0.034	0.030	0.012	0.014
Methylhistidine	−0.006	−0.001	—	−0.027	—
Ammonia	0.009	—	—	0.005	—
Taurine	—	0.003	—	—	0.001
Threonine	—	−0.003	—	—	—
Phosphoethanolamine	—	—	—	—	0.012
Gender (male)	0.554	—	—	0.182	—
Caucasian	0.110	—	—	—	—

**Table 4:** Predictive model performance.

Performance Index	Model 1		Model 2		Model 3		Model 4		Model 5		Training		Test	
	Training	Test	Training	Test	Training	Test	Training	Test	Training	Test	Average	SD	Average	SD
True positives	44	17	42	19	43	17	48	22	48	23	45.0	2.8	19.6	2.8
True negatives	13	8	18	6	17	7	7	1	5	3	12.0	5.8	5.0	2.9
False negatives	3	7	5	5	4	7	0	1	0	0	2.4	2.3	4.0	3.3
False positives	20	8	15	10	16	9	25	16	27	14	20.6	5.3	11.4	3.4
Sensitivity	0.936	0.708	0.893	0.792	0.915	0.708	1.000	0.957	1.000	1.000	0.949	0.049	0.833	0.138
Specificity	0.394	0.500	0.545	0.375	0.515	0.438	0.219	0.059	0.156	0.176	0.366	0.174	0.310	0.186
Accuracy	0.713	0.625	0.750	0.625	0.750	0.600	0.688	0.575	0.663	0.650	0.713	0.038	0.615	0.029
ROC AUC	0.832	0.659	0.845	0.578	0.802	0.570	0.756	0.724	0.771	0.703	0.801	0.038	0.647	0.071

## Discussion

This study investigated the possibility of establishing a model to predict acamprosate treatment response in alcohol dependent patients utilizing demographics, alcohol use history, clinical assessments and serum metabolite levels. Overall, our findings suggest that combining baseline serum metabolites and clinical scores as variables in a multivariate model provides some predictive power to differentiate responders and non-responders to 3 months of acamprosate treatment. However, the mean AUC of 0.65 and the poor specificity of the predictive models, suggests that studies with larger sample sizes that explore more potential biomarkers are needed to expand the clinical utility of predictive algorithms for pharmaceutical response in AUD. To our knowledge, this is the first study that evaluates the potential accuracy of predictive models that incorporate metabolomic biomarkers and clinical factors to determine treatment response to acamprosate. This approach could potentially be used to predict response to other FDA-approved medications as well as novel drugs to treat AUD.

We found that the average sensitivity was high in the training (94.9%) and the test (83.3%) samples. A high sensitivity suggests that this model is very good at detecting true positives or those that are predicted to respond and do respond to acamprosate. From a clinical perspective, identifying those patients that are predicted to respond would allow the patient to get the best medication for them at the beginning of treatment. This could dramatically improve the ways that patients with AUD are treated. The low specificity indicates that the model is unable to identify non-responders. Caution should be noted as, if too many people are predicted to “respond” among eventual non-responders, they may be given a treatment from which they may not benefit. However, given how few pharmacological treatment options are available for AUDs, we believe that in this context high sensitivity is more important than high specificity, i.e. we are willing to accept some false positives – patients who are predicted to respond but eventually do not respond to acamprosate treatment – in order to correctly identify most patients that are likely to respond to this treatment. Nevertheless, to be clinically useful a predictive model should have high sensitivity and at least moderately high specificity. The low specificity in our study may partly reflect over-fitting and is also due to the difference in responder/non-responder proportions (i.e. data imbalance). In our random split of the sample into training and test data (which was done 5 independent times), we performed a stratified random split, so that the ratio of responders to non-responders would be similar in all the training and test subsets. Because in the complete sample the number of responders is higher than non-responders (59% vs. 41%), each training and test set also had this unbalanced number of cases and controls.

Our models had an average area under a receive operating characteristic (ROC) curve of 0.80 in the training samples and an AUC of 0.65 in the test samples. Prospective studies using a larger sample are necessary to identify new models with improved predictive power. A novel aspect of our model is the inclusion of serum metabolites as predictors. Using metabolomics to aid in prescription of medications is feasible since metabolomics can be ordered as a clinical test used to evaluate a patient’s pretreatment metabolite signature prior to selection of a pharmacological agent^29^, but standardization of the method between laboratories is critical.

We found that baseline PACS and aspartate levels were the two variables that consistently were associated with acamprosate treatment response in all 5 models. Lower craving scores have been reported by our previous study to be associated with acamprosate treatment response^28^. Furthermore, our group has also found that increased alcohol craving is associated with shorter abstinence^18^. These data suggest that patients with low craving for alcohol may be more likely to be treated with acamprosate or abstain in general from alcohol consumption. On the other hand, data from the COMBINE study was reanalyzed and it was found that acamprosate was most effective in patients with shorter abstinence (1 week or less) length before initiation of treatment^30^. Although this is not a direct assessment of alcohol craving, it suggests that a patient with lower craving that has been abstinent from 1 week or less may be the ideal candidate for treatment. Previously, we found that acamprosate treatment response was associated with elevated baseline glutamate levels^28^. In addition, several studies have identified genetic associations related to glutamate signaling with acamprosate treatment response^16–18^. In the present study, we found that aspartate and glutamate levels were significantly elevated in responders to acamprosate and associated with acamprosate treatment response, although aspartate was the only amino acid significantly associated with acamprosate response in all 5 predictive models. Interestingly, aspartate can be converted to glutamate via aspartate transaminase (AST) in serum. AST is frequently used as a marker of liver function and has been used as a marker of alcohol use. Future studies that incorporate metabolomic, genetic, and clinical characteristics as variables may reveal better prospective predictive models that could be used to predict acamprosate treatment response.

Our findings should be considered in light of the study’s limitations. It is important to emphasize that the metabolomic variables considered in this investigation were collected from serum, which may not directly reflect the metabolite levels in the brain. Future studies should be conducted to evaluate baseline brain metabolite level differences between responders and non-responders using non-invasive techniques such as magnetic resonance spectroscopy (MRS)^31–33^, and establish the relationship between blood and brain metabolites, so that using blood metabolites, which is more cost-effective, as proxy to those in the brain could be justified. On the other hand, a baseline serum metabolite profile provides a more feasible clinical test to screen for potential response type than that from an MRS scan. In addition, the clinical features and the types of metabolites we used in the development of the predictive models are vast yet limited. Further studies using global metabolomics, which covers all the detectable metabolites may lead to stronger predictive models to predict acamprosate treatment response. In addition, based on recent finding that the active component of acamprosate might be calcium^34^, calcium-regulating metabolites may play a role in determining acamprosate treatment response. However, global metabolomics approaches are only semi-quantitative. Finally, although the quantification of metabolite concentrations using LC/MS/MS is based on standard concentration curves and each sample is spiked with an internal standard for normalization between sample runs, it is possible that concentrations of metabolites may vary between analysis batches or institutions. Thus, the use of metabolite ratios may allow consistency between analysis batches or institutions.

In conclusion, our study highlights the use of metabolic and clinical biomarkers to create a predictive model to determine whether or not any single patient with AUD has a good probability of responding to acamprosate. This study takes us one step closer to a personalized treatment approach for patients with AUD.

## Methods

### Subjects

The study was conducted in compliance with the Code of Ethics of the World Medical Association (Declaration of Helsinki) and was approved by the Institutional Review Board of Mayo Clinic (IRB number: 07-007204); all participants provided informed consent. We utilized metabolomics data from a subset of 120 subjects recruited as part of a previously described study^18, 28^. All participants met DSM-IV-TR criteria for alcohol dependence. A detailed description of inclusion and exclusion criteria used for this study has been published previously^28^. Recruitment of subjects was from community based residential and outpatient treatment programs affiliated with Mayo Clinic in Rochester, Minnesota and the Mayo Clinic Health System sites in Austin, Minnesota, Albert Lea, Minnesota and La Crosse, Wisconsin as previously described^18, 28^. In total, 443 subjects were assessed for eligibility and acamprosate was initiated. Metabolomics assays were performed on a subset of 120 subjects with complete demographic and clinical data, including alcohol use data at 12-week follow-up, and available serum samples. This group of 120 included 71 subjects that remained completely abstinent during the 12 weeks of acamprosate treatment (“treatment responders”) and 49 subjects that relapse (any alcohol consumption; “non-responders”).

### Acamprosate treatment and monitoring

Participants were prescribed one 333 mg tablet three times a day for the first week to determine tolerance of medication. Then, a standard dose of two 333 mg tablet three times a day was prescribed. Subjects were followed monthly for 3 months by in-person interviews (the first and third months) and by phone (the second month) to obtain accurate sobriety, medication compliance (pill counts) and presence of psychiatric symptoms. Response to acamprosate was defined as no alcohol consumption during the 3-month treatment period while non-response was defined as any amount of alcohol consumption during the 3-month treatment period. Response vs. non-response outcome was determined by self-report (TLFB-30) and by measuring levels of GGT to assess accuracy of self-report.

### Clinical assessments

Prior to the start of acamprosate treatment (baseline), self-reported history of alcohol consumption was collected by a research coordinator *via* the 30-day Timeline Follow Back (TLFB-30) method^35^. Baseline anxiety and depressive symptom severity were assessed by the 7-item General Anxiety Disorder Questionnaire [GAD-7; ref. 36] and the 9-item Patient Health Questionnaire [PHQ-9^37^]. Baseline alcohol craving was assessed by the Pennsylvania Alcohol Craving Scale [PACS; ref. 38]. The PACS is a five-item self-report measure with each question scaled from 0 to 6. It quantifies the frequency, intensity, duration of alcohol craving, and the ability to resist drinking over the previous week. Higher scores indicate stronger craving.

### Serum collection

At baseline, approximately 20 ml of blood was collected from each subject, which usually occurred between noon and 3PM. Blood was collected to analyze metabolite levels as well as gamma-glutamyl transpeptidase (GGT) levels as a biological marker of alcohol consumption. Venipuncture was performed using standard techniques. All tubes were labeled with a study identifier, collection date, and time of draw. After collection, samples were electronically accessioned at the Biospecimens Accessioning Processing (BAP) facility at Mayo Clinic. Samples were subsequently spun down for 15 min at 2900 × g at 4 °C and serum was aliquoted into 250 μl samples and stored at −80 °C within 2 h to minimize any possible metabolite degradation. All serum samples were thawed on ice for approximately 2 h before use. Glutamine synthetase activity was measured as described^39^.

### Metabolomics using LC-MS/MS

The metabolomics method for analyzing these serum samples using LC-MS/MS has been described previously^28^. Briefly, serum amino acid calibration standards were prepared with a MassTrak Amino Acid Analysis Solution (AAA) kit (Waters Corp, Milford, MA) according to instructions with slight modifications for detection on a mass spectrometer^40^. To measure accurate metabolite concentrations, we first normalized each sample to the internal standard. Serum samples of 10 μl were spiked with an internal standard then derivatized according to MassTrak instructions. The amino acid derivatizing reagent used was 6-aminoquinolyl-N-hydroxysuccinimidyl carbamate. Then, concentrations based on the standard curve were determined. A 10-point standard concentration curve was made from the calibration standard solution to calculate amino acid concentrations in serum samples. High resolution separation was done using an Acquity UPLC system and injecting 1 ml of derviatized solution, with a UPLC BEH C18 column (Waters Corp, Milford, MA). Mass detection was completed on a TSQ Ultra Quantum running in ESI positive mode (Thermo Scientific, Waltham, MA).

### Statistical analysis

Prior to building multivariable predictive models of acamprosate response, we compared baseline demographic, clinical, and metabolomic features between responders (*n* = 71) and non-responders (*n* = 49). Unpaired *t* test were used for continuous variables, while chi-square tests were used for categorical variables.

To investigate the potential utility of multivariable prediction of acamprosate treatment response, subjects were randomized into a training set (2/3 of subjects; *n* = 80) and a test/validation set (1/3 of subjects; *n* = 40); this random split was repeated 5 times to allow assessment of variability in the performance of the predictive models. The random split of subjects into test and training datasets was performed in responder/non-responder strata, to ensure a similar proportion of responders across training and test subsets. Sparse logistic regression based on the Lasso approach with four-fold cross-validation optimized for the model deviance was then used for model building, beginning with all variables shown in Tables 1 and 2. The analysis was implemented using the cv.glmnet function in the glmnet R package (https://cran.r-project.org/web/packages/glmnet/glmnet.pdf), with nfolds = 4 and the type = “deviance” option. The models built in the training sets were then used to predict response in the corresponding independent test sets, and the area under the Receiver Operating Characteristic (ROC) curve (AUC), as well as the sensitivity and specificity based on a 0.5 predicted probability cut-off, were calculated to evaluate model performance in the test sets. Mean imputation of five of the metabolic predictors (predictor ([% missing]: phosphoethanolamine [6.7], 1-methylhistidine [17.5], 3- methylhistidine3 [5.8], β-aminoisobutyric-acid [9.2], cysthationine [5.8]) was conducted prior to analysis to preserve the sample size and create the complete data needed for the Lasso models.
